# Capillary refill time during fluid resuscitation in patients with sepsis-related hyperlactatemia at the emergency department is related to mortality

**DOI:** 10.1371/journal.pone.0188548

**Published:** 2017-11-27

**Authors:** Barbara Lara, Luis Enberg, Marcos Ortega, Paula Leon, Cristobal Kripper, Pablo Aguilera, Eduardo Kattan, Ricardo Castro, Jan Bakker, Glenn Hernandez

**Affiliations:** 1 Programa de Medicina de Urgencia, Facultad de Medicina, Pontificia Universidad Católica de Chile, Santiago, Chile; 2 Departamento de Medicina Intensiva, Facultad de Medicina, Pontificia Universidad Católica de Chile, Santiago, Chile; 3 Erasmus MC University Medical Center, Dept. Intensive Care Adults, Rotterdam, CA, The Netherlands; 4 Division of Pulmonary, Allergy, and Critical Care Medicine, Department of Medicine, Columbia University Medical Center, New York, NY, United States of America; 5 Division of Pulmonary, and Critical Care Medicine, New York University—Langone, New York, NY, United States of America; Azienda Ospedaliero Universitaria Careggi, ITALY

## Abstract

**Introduction:**

Acute circulatory dysfunction in patients with sepsis can evolve rapidly into a progressive stage associated with high mortality. Early recognition and adequate resuscitation could improve outcome. However, since the spectrum of clinical presentation is quite variable, signs of hypoperfusion are frequently unrecognized in patients just admitted to the emergency department (ED). Hyperlactatemia is considered a key parameter to disclose tissue hypoxia but it is not universally available and getting timely results can be challenging in low resource settings. In addition, non-hypoxic sources can be involved in hyperlactatemia, and a misinterpretation could lead to over-resuscitation in an unknown number of cases. Capillary refill time (CRT) is a marker of peripheral perfusion that worsens during circulatory failure. An abnormal CRT in septic shock patients after ICU-based resuscitation has been associated with poor outcome. The aim of this study was to determine the prevalence of abnormal CRT in patients with sepsis-related hyperlactatemia in the early phase after ED admission, and its relationship with outcome.

**Methods:**

We performed a prospective observational study. Septic patients with hyperlactemia at ED admission subjected to an initial fluid resuscitation (FR) were included. CRT and other parameters were assessed before and after FR. CRT-normal or CRT-abnormal subgroups were defined according to the status of CRT following initial FR, and major outcomes were registered.

**Results:**

Ninety-five hyperlactatemic septic patients were included. Thirty-one percent had abnormal CRT at ED arrival. After FR, 87 patients exhibited normal CRT, and 8 an abnormal one. Patients with abnormal CRT had an increased risk of adverse outcomes (88% vs. 20% p<0.001; RR 4.4 [2.7–7.4]), and hospital mortality (63% vs. 9% p<0.001; RR 6.7 [2.9–16]) as compared to those with normal CRT after FR. Specifically, CRT-normal patients required less frequently mechanical ventilation, renal replacement therapy, and ICU admission, and exhibited a lower hospital mortality.

**Conclusions:**

Hyperlactatemic sepsis patients with abnormal CRT after initial fluid resuscitation exhibit higher mortality and worse clinical outcomes than patients with normal CRT.

## Introduction

Early recognition and adequate resuscitation of patients with sepsis associated circulatory dysfunction is a fundamental challenge at the emergency department (ED) [1]. However, the clinical picture ranges from difficult recognizable abnormalities in peripheral perfusion to advanced septic shock with circulatory collapse [2, 3].

Although hyperlactatemia is still considered a key parameter to disclose tissue hypoxia and trigger resuscitation, [4–6], other factors than hypoperfusion have been associated with sepsis-related hyperlactatemia. In the early hours of resuscitation lactate decreases in parallel with normalization of peripheral perfusion and other parameters of regional blood flow [7]. Therefore, resuscitation based only on persisting hyperlactatemia might be inappropriate [8].

During circulatory dysfunction, compensatory mechanisms redistribute flow away from non-vital regions like the skin, making this a window for clinical assessment of circulatory dysfunction [2, 3, 9, 10]. Parameters of peripheral perfusion can be easily evaluated at the bedside [11] and are used as triggers for fluid resuscitation (FR) in patients with sepsis-related acute circulatory dysfunction [12, 13]. In addition, persistent abnormal peripheral perfusion has been associated with increased morbidity and mortality in patients with sepsis and septic shock [12–16] after intensive care resuscitation but this has not been explored at earlier stages.

Therefore, we studied the prevalence of an abnormal capillary refill time (CRT) in hyperlactatemic patients with sepsis just admitted to the ED. We hypothesized that abnormal CRT after FR would prove to be a good risk stratification parameter to take into consideration when assessing hyperlactatemic septic patients during very early resuscitation.

## Methods

### Study design and setting

We conducted a prospective observational study at the ED of the University Hospital of the Pontificia Universidad Católica de Chile. A mean of 3,500 adult patients are treated monthly at this ED of whom approximately 13% are hospitalized. The Institutional Review Board of the Facultad de Medicina, Pontificia Universidad Católica de Chile approved the study (CEC-MEDUC 14–379; August 5, 2014), and waived the requirement of informed consent since the study design did not deviate from best standard of care.

### Selection of participants

All consecutive adult patients admitted to the ED for sepsis were considered eligible for this study. Only those with hyperlactatemia (lactate ≥ 2mg/dl) at ED arrival were included for the analysis. Sepsis required a documented or suspected source of infection and evidence of systemic inflammation [4]. We excluded patients that received fluids before ED arrival, patients in whom CRT could not be assessed (Raynaud syndrome, severe hypothermia), patients that required a peremptory surgical procedure in the first hour, or had a Do-Not-Resuscitate (DNR) order.

All patients with suspected sepsis were treated following a local standard protocol, which has been adapted from Surviving Sepsis Campaign (SSC) guidelines [4]. Briefly, upon diagnosis of sepsis patients were subjected to: 1) Basal evaluation of vital signs, and peripheral venous catheters insertion for sampling, and drugs or fluid administration; 2) Thorough search for infectious sources including clinical examination, laboratory testing, blood and clinically-oriented local cultures, and images as required; 3) Administration of IV antibiotics during the first hour depending on the suspected source and organism; 4) An initial FR with a volume up to 30 ml/kg of normal saline administered in 2 h. The physician in charge determined the volume and maximal rate of the fluids according to basic hemodynamic parameters and individual risk assessment; 5) Standardized assessment of the circulation including serum lactate levels before and after this initial FR as recommended by the SSC [4]. Patients were then transferred to the intensive Care Unit (ICU), step-down units, or wards depending on individual decisions.

### Methods and measurements

#### Peripheral perfusion assessment

All residents and staff physicians were trained to assess peripheral perfusion before the start of the study. CRT was measured by applying firm pressure to the distal phalanx of the right index finger for 10 seconds. Time for return of the normal color at the ventral surface was recorded with a mobile phone chronometer, and 3 seconds was defined as the upper normal limit. The cut-off value for CRT was selected based on our previous study in septic shock patients in whom a CRT <4 s predicted 85% hospital survival rate and lactate normalization [13], and the study of Ait-Oifella et al who demonstrated that a threshold of index CRT at 2.4 s predicted 14-day outcome with good sensitivity and specificity [16].

Only trained physicians collected CRT measurements. Their inter-observer reliability was assessed after the training and before the study start date [16]. CRT was assessed immediately before and after FR.

Collection of chart data and follow up measurements in the ICU, step-down unit or wards, as well as statistical analyses were made by investigators not involved in the direct clinical care of these patients.

#### Outcomes

The specific prospective data set for this study included baseline clinical and vital signs, demographic and infection-related data, severity scores (acute and chronic health evaluation score II (APACHE II) [17], and sequential organ failure assessment score (SOFA) [18]), and perfusion assessments before and after the first FR. Follow-up dataset included second day-SOFA, need and length of mechanical ventilation (MV), ICU and hospital length of stay, and hospital mortality.

We also defined a composite outcome that included: ICU length of stay ≥72 hours, need for MV ≥48 hours, need of renal replacement therapy, or hospital mortality.

#### Analysis

We decided on CRT status after FR as the primary variable to be analyzed, and performed secondary analyses on global hemodynamics, lactate, and their relationship with major outcomes. We planned to collect data on 100 consecutive patients that met the inclusion criteria in this observational study.

All studied variables were tested for normal distribution with Kolmogorov-Smirnov test. Patient characteristics are reported as mean ± standard deviation, median (25th–75th percentiles) for non-normal distributions, and percentages as appropriate.

Intra-group comparisons were performed using t-test for repeated measures, chi-square test, or Wilcoxon signed-rank test. Between-group comparisons were reported as relative risk or mean/median differences and tested using t-test, chi-square test, Fisher’s exact test, and Mann-Whitney test as appropriate.

A p-value < 0.05 was considered as statistically significant. All reported p-values are two sided. All analyses were performed using STATA version 13.1.

## Results

### Characteristics of the study subjects

A total of 60,571 patients were treated at the ED during the study period, of which 9,972 (16%) were admitted to the hospital, including 323 patients with a diagnosis of infection and some organ dysfunction. The study was stopped after 100 consecutive patients fulfilling inclusion criteria. Five patients were later excluded because they met exclusion criteria. Demographic and baseline data of this cohort is shown in Table 1.

**Table 1:** Clinical and demographic data of the study population.

Variables	All patients (N = 95)
Age (y)	67 ± 18
Male (%)	47 (49)
Past Medical History	
Diabetes (%)	26 (27)
Hypertension (%)	48 (50)
Chronic renal failure (%)	16 (17)
Heart failure (%)	10 (11)
Liver failure (%)	5 (5)
Immunosuppression (%)	14 (15)
Cancer (%)	23 (24)
COPD (%)	5 (5)
Sepsis source	
Abdominal (%)	33 (34)
Respiratory (%)	31 (33)
Renal/urologic (%)	21 (22)
CNS (%)	4 (4)
Skin (%)	3 (3)
Other (%)	3 (3)
APACHE II [IQR]	16 [10–21]
SOFA day 1 [IQR]	4 [2–7]
SOFA day 2 [IQR]	3 [1–6]

COPD: Chronic obstructive pulmonary disease; CNS: Central nervous system; APACHE: Acute physiology and chronic health evaluation; SOFA: Sequential organ failure assessment. Values are expressed as n (percentage), median [interquartile range] or mean ± SD.

The mean lactate level at ED presentation was 4.2 mmol/l. Physiologic parameters before and after FR are shown in Table 2.

**Table 2:** Physiologic parameters pre- and post-fluid resuscitation.

Parameter	Pre fluid resuscitation	Post fluid resuscitation
Heart Rate (bpm)	110 ± 21	98 ± 15
Systolic blood pressure (mmHg)	120 ± 30	115 ± 27
Mean arterial pressure (mmHg)	84 ± 20	80 ± 16
Respiratory rate (bpm)	27 ± 8	23 ± 6
Temperature (C°)	38 ± 1	37 ± 1
Capillary refill time (seconds)	3 [2–4]	2 [1–3]
Lactate (mmol/L)	4.3 ± 2.5	3.0 ± 2.8

Values are expressed as median [interquartile range] or mean ± SD

**Fig 1:**
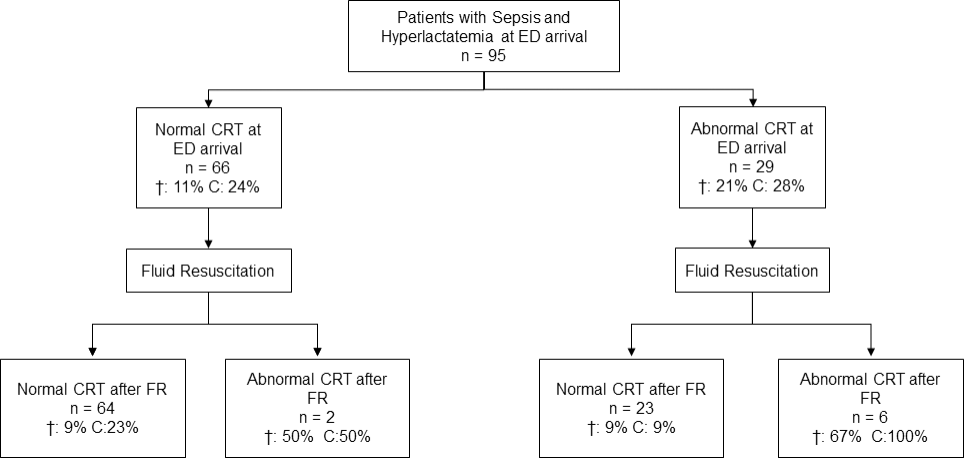
Distribution and outcomes of hyperlactatemic septic patients, according to capillary refill time before and after initial fluid resuscitation. ED: Emergency Department; CRT: Capillary refill time; FR: fluid resuscitation; C: Composite outcome of ICU length of stay ≥72 hours, need for MV ≥48 hours, need of renal replacement therapy, or hospital mortality.

The distribution of patients with normal versus abnormal CRT before and after FR, and their outcome is shown in Fig 1.

Basal CRT status did not predict outcome. In contrast, patients with abnormal CRT after initial fluid resuscitation had an increased risk of adverse outcomes (88% vs. 20% p<0.001; RR 4.4 [2.7–7.4]) and hospital mortality (63% vs. 9% p<0.001; RR 6.7 [2.9–16]) as compared with normal CRT. Moreover, the maintenance of an abnormal CRT after FR, was also associated with a higher rate of adverse outcomes (Table 3).

**Table 3:** Physiologic parameters and outcomes of patients with abnormal CRT at admission according to CRT status after initial fluid resuscitation.

Physiologic parameters after FR and outcomes	Normal CRT After FR N:23	Abnormal CRT after FR N:6	p-value
Heart rate (bpm)	97 ± 14	99 ± 23	0.91
Systolic blood pressure (mmHg)	122 ± 30	101 ± 26	0.22
Mean arterial pressure (mmHg)	83 ± 20	76 ± 23	0.45
Respiratory rate (bpm)	24 ± 5	23 ± 7	0.48
Temperature (C°)	37 ± 1	36 ± 0.7	0.05
Capillary refill time (seconds)	2.3 ± 0.6	6.1 ± 3.2	<0.01
Lactate (mmol/L)	3.4 ± 2.4	11 ± 7	<0.01
SOFA day 1	4[2–6]	9[9–10]	<0.01
MV	2 (9%)	4 (67%)	<0.01
MV [days]	2.5 [1–4]	2.5 [1–15.5]	0.80
ICU admission	4 (17%)	5 (83%)	<0.01
ICU length of stay (days)	2[1.5–5.5]	6[1–20]	0.64
Need of RRT	0 (0%)	2 (33%)	0.04
Hospital length of stay (days)	9[5–11]	12.5[1–35]	0.53
Hospital mortality	2 (9%)	4 (67%)	<0.01

Values are expressed as n (percentage), median [interquartile range] or mean ± SD.

SOFA: Sequential organ failure assessment; MV: Mechanical ventilation; RRT: Renal replacement therapy.

Seventy patients (73%) normalized or decreased lactate levels ≥ 20% after FR. Patients with normal CRT after FR were more likely to normalize or decrease lactate levels than those with abnormal CRT (77 vs. 38%, p = 0.01).

## Discussion

Our main finding is that patients with sepsis and hyperlactatemia exhibiting normal CRT after the very first fluid resuscitation in the ED have a low morbidity and mortality risk. In contrast, abnormal peripheral perfusion despite initial FR is a strong predictor of worse outcome. In any case, this finding could have important implications in triage decisions for intensive care admission.

Of the classic three windows for clinical perfusion assessment proposed by Weil [3], peripheral perfusion appears as particularly attractive. Indeed, the skin lacks auto-regulatory flow control, and therefore the activated sympathetic nervous system decreases local perfusion during acute circulatory dysfunction [3, 19]. This process can be evaluated by assessment of peripheral perfusion via fast and simple clinical techniques. The concept of CRT evaluation is based on this assumption. It was proposed initially in trauma patients [20], but when subsequent studies did not find a clear correlation with systemic hemodynamics it was largely abandoned [21]. More recently though, Lima et al found that abnormal peripheral perfusion was associated with hyperlactatemia and organ dysfunctions in critically ill patients [14]. Since then, a robust body of evidence that supports the strong prognostic value of abnormal peripheral perfusion in the ICU context has been published [12–16]. Nevertheless, most of the data were collected after extensive ICU-based resuscitation and thus might have limited applicability at the ED or pre-ICU levels. Therefore, our study adds an important piece of information. Abnormal peripheral perfusion post-initial FR might predict a more complex circulatory dysfunction very early after ED admission in septic patients with hyperlactatemia.

Beyond its prognostic value, some recent data provide a dynamic view of peripheral perfusion response to resuscitation in septic shock patients. Hernandez et al showed that CRT was the first parameter to normalize after fluid loading in the ICU setting, thus opening research pathways on peripheral perfusion as a potential resuscitation target [13]. More provocatively, van Genderen et al performed a randomized controlled trial in 30 ICU patients comparing two resuscitation strategies [22]. The study demonstrated that using normal peripheral perfusion to limit additional fluid resuscitation is safe, and associated with a less positive fluid balance and improved organ function.

Defining sepsis-related acute circulatory dysfunction early after ED admission is still a grey zone. Indeed, no clear definition for this early phase has been agreed, and the presence of hypotension, hyperlactatemia, and abnormal peripheral perfusion are frequently used in clinical practice as triggers for fluid resuscitation [12, 13]. The SSC’s bundles are targeted to patients with persistent hypotension following FR and/or lactate levels ≥ 4 mmol/ [4]. The concept is however flawed by the assumption that high lactate levels mostly result from tissue hypoperfusion, a model that is still a matter of debate [2, 8, 23]. As the clinical spectrum of acute circulatory dysfunction is broad, both the recent SSC bundle’s amendment [24] and the 2014 European Consensus on circulatory shock [6] recommend assessment of peripheral perfusion to evaluate response. In this sense, our results add important information about the prognostic relevance of an abnormal peripheral perfusion very early on the resuscitation process in hyperlactatemic septic patients.

Peripheral perfusion can be assessed by different techniques [11]. Nevertheless, we preferred to focus our study on a parameter easily measurable in almost any condition with even minimal training. CRT has been used in many clinical studies, most confirming the association between prolonged CRT and increased morbidity and mortality [13, 14, 16, 25]. However, CRT assessment is susceptible to factors that can deeply affect the results, such as environmental, skin and core temperatures, age, ambient light conditions, and the duration, amount and site of pressure application [26]. Some of these conditions can be controlled and the measurements standardized to reduce ambient-related and technical variability.

The issue of inter-observer reliability has also been raised [27–30], but recent data support a good agreement when observers are previously trained with standardized procedures [14, 25]. van Genderen et al explored inter-observer reliability in CRT assessment between different health-care workers demonstrating a good overall agreement [25]. In another study, CRT exhibited a good correlation with objective variables of peripheral perfusion [26]. On the other hand, Ait-Oufella et al, demonstrated that CRT is highly reproducible in a prospective cohort of septic shock patients with an excellent inter-rater concordance [16]. In our opinion, difficulties in implementing routine CRT assessment can be overridden by education, training and standardization of the technique, and by reducing the impact of ambient-related factors, as we did in this study.

This study has several limitations. The limited number of patients and the single center setup warrants confirmation in a larger multicenter study. The low mortality of the study population is probably explained by the fact that the study was performed in a university hospital where patients tend to consult earlier resembling reports from similar contexts [31, 32]. We did not assess inter-observer reliability because it was out of scope for our purposes. We cannot assure that initial fluid resuscitation at the ED completely optimized preload status in our patients, and in fact some patients were transferred to the ICU for further resuscitation with more advanced monitoring including fluid-responsiveness assessment. Finally, we cannot assure that implementing a CRT-targeted resuscitation in hyperlactatemic septic patients could have changed the high mortality in the CRT-abnormal subgroup, but we strongly believe that its evaluation can add another important variable for early risk stratification at the ED.

## Conclusions

Patients with sepsis and hyperlactatemia with an abnormal CRT after the first fluid resuscitation in the ED exhibit significantly worse outcomes when compared to patients with normal CRT. As CRT is a simple and almost universally available tool, these findings, if confirmed by future studies, could be a valuable contribution for the triage, treatment, and follow-up of patients with sepsis-related acute circulatory dysfunction in the early resuscitation context.

## Supporting information

S1 Database Database of patients. (XLS)

## Abbreviations

ED: emergency department
ICU: intensive care unit
FR: fluid resuscitation
SSC: Surviving Sepsis Campaign
CRT: capillary refill time
APACHE II: acute and chronic health evaluation score II
SOFA: sequential organ failure assessment score
MV: mechanical ventilation
